# Nanoscale ultrafast camera unveils light-matter dynamics in real time and space

**DOI:** 10.1038/s41377-025-01908-9

**Published:** 2025-06-20

**Authors:** Jae Won Ryu, Kyoung-Duck Park

**Affiliations:** 1 Department of Physics and Department of Semiconductor Engineering, Pohang University of Science and Technology (POSTECH), Pohang, 37673 Republic of Korea; 2 Department of Mechanical Engineering, Pohang University of Science and Technology (POSTECH), Pohang, 37673 Republic of Korea; 3 Center for Multidimensional Carbon Materials (CMCM), Institute for Basic Science (IBS), Ulsan, 44919 Republic of Korea; 4 Institute for Convergence Research and Education in Advanced Technology, Yonsei University, Seoul, 03722 Republic of Korea

**Keywords:** Optics and photonics, Optical physics

## Abstract

Ultrafast tip-based microscopy has evolved to meet three critical parameters in optical characterization: spatial, spectral, and temporal resolution. This advancement provides deep insights into light-matter interactions in both real time and real space. In particular, it allows direct observation of polaritons, quantum states, and nonequilibrium dynamics, especially in low-dimensional quantum materials.

## Abstract (web summary)

The integration of ultrafast spectroscopy with scanning probe microscopy realizes direct visualization of femtosecond-scale light-matter interactions beyond the diffraction limit.

## Graphical abstract

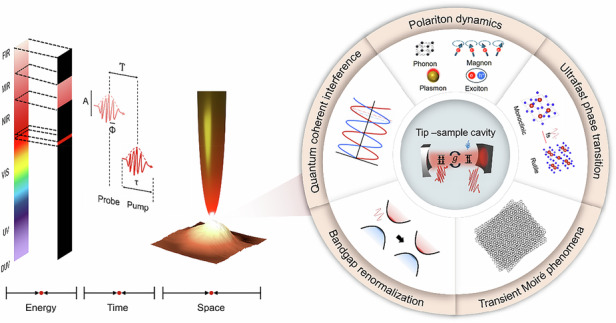

Optical probing has long been a cornerstone of material characterization, revealing fundamental electronic, vibrational, and optical properties. Its versatility has made it indispensable across disciplines, fueling breakthroughs from basic science to advanced engineering. Yet, as research delves deeper into nanoscale phenomena, conventional optical techniques encounter a fundamental challenge: the diffraction limit. Governed by Abbe’s principle^1^, this constraint caps spatial resolution at roughly half the wavelength of light, hindering the ability to explore structures at the smallest scales.

To overcome this limitation, optical techniques have been integrated with scanning probe microscopy, leading to advancements, such as tip-enhanced photoluminescence^2^, tip-enhanced Raman spectroscopy^3,4^, and scattering-type scanning near-field optical microscopy (*s*-SNOM)^5^. These approaches leverage the material^6^ and geometric^7^ properties of nanoscale tips in distinct ways to enhance local optical fields and detect elastic^8,9^ and inelastic^10,11^ near-field scattering signals. This enables deep subwavelength imaging while allowing precise control over light-matter interactions at the nanoscale^12–16^. However, conventional tip-based methods primarily capture static material properties, making it challenging to probe transient many-body dynamics^17^ and coherent light-matter interactions^18^. Unlocking the ultrafast regime requires a fundamentally new approach.

A recently published review in eLight introduces state-of-the-art ultrafast tip-based nano-spectroscopy and nano-imaging techniques designed to push beyond traditional resolution limits^19^. As illustrated in Fig. 1, these methods integrate pump-probe spectroscopy with tip-based microscopy, enabling direct measurement of transient optical and electronic phenomena with femtosecond precision by tuning the pump-probe delay. This spatiotemporally resolved approach allows direct visualization of quantum excitations, polariton propagation, and ultrafast carrier dynamics. The reveiw highlights three cutting-edge advances in this field: ultrafast *s*-SNOM, ultrafast nanofocusing, and ultrafast STM, demonstrating the power of ultrafast tip-based microscopy in resolving dynamic nanoscale phenomena with unprecedented precision.

**Fig. 1:**
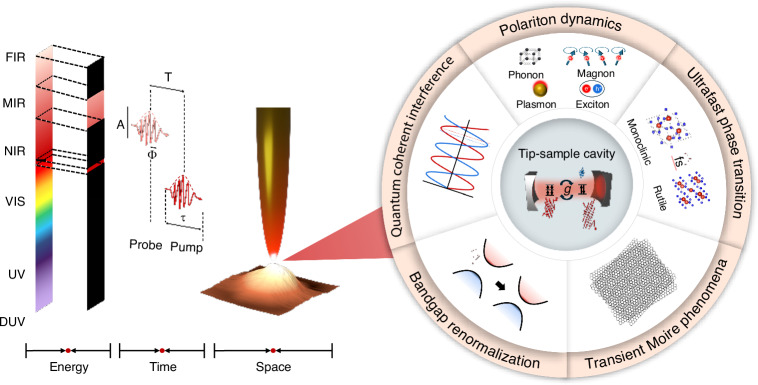
Illustration of ultrafast tip-based nano-spectroscopy and its applications

Ultrafast *s*-SNOM is an advanced near-field imaging technique that isolates pure near-field signals through interferometric filtering^20^, which enables high-resolution visualization of ultrafast light-matter interactions. Depending on the properties of the light source, it can be utilized for techniques ranging from monochromatic scanning to broadband scattering spectroscopy. This capability makes it particularly effective for probing spatial heterogeneity in polaritons, excitons, and phase transitions, especially in the mid- to far-infrared spectral range, where vibrational and electronic excitations govern ultrafast material dynamics.

Ultrafast nanofocusing overcomes the diffraction limit by adiabatically compressing broadband ultrafast pulses into deep-subwavelength plasmonic modes^21,22^. This approach fundamentally relies on a specialized tip fabrication process to couple incident light into the tip, generating a background-free, tightly confined optical hotspot at the tip apex. The symmetry of the tip system, combined with a high-power pulsed laser, harnesses its potential for nonlinear spectroscopy and coherent electron dynamics. Additionally, its application as an ultrafast electron point projection source further broadens its functionality, offering vast opportunities for expansion.

Unlike optical near-field techniques, ultrafast STM directly measures tunneling currents modulated by ultrafast laser excitation, making it a powerful tool for probing coherent electron motion, many-body interactions, and light-driven phase transitions with atomic-scale precision^23,24^. By integrating THz and optical pulse-driven tunneling, it enables the capture of sub-cycle carrier dynamics, offering direct access to electronic wavefunctions and ultrafast transport phenomena. The ability to resolve electronic states with femtosecond time resolution and sub-angstrom spatial resolution allows ultrafast STM to probe quantum coherence, charge localization, and transient excitations at the single-atom level. Furthermore, advances in phase-sensitive detection and pump-probe synchronization continue to expand its capabilities, opening new avenues for studying nonequilibrium quantum phenomena in low-dimensional and strongly correlated systems.

Ultrafast tip-based microscopy offers a versatile platform for probing nanoscale light-matter interactions across a broad range of timescales, spatial resolutions, and spectral domains. Past experiments have demonstrated the adaptability of ultrafast s-SNOM, ultrafast nanofocusing, and ultrafast STM for different material systems and physical phenomena across diverse spatial, temporal, and spectral ranges. This scalability makes ultrafast tip-based techniques indispensable for studying diverse ultrafast processes, from quantum excitations to carrier transport, with tailored resolutions optimized for specific applications. Advances in detection schemes and experimental configurations will continue to push these limits, enhancing both precision and applicability. By leveraging this flexibility, ultrafast tip-based microscopy is poised to unlock new frontiers in nanoscale optical characterization and ultrafast material dynamics.
